# Predictive modeling of initiation and delayed mental health contact for depression

**DOI:** 10.1186/s12913-024-10870-y

**Published:** 2024-04-25

**Authors:** Vanessa Panaite, Dezon K. Finch, Paul Pfeiffer, Nathan J. Cohen, Amy Alman, Jolie Haun, Susan K. Schultz, Shannon R. Miles, Heather G. Belanger, F. Andrew F. Kozel, Jonathan Rottenberg, Andrew R. Devendorf, Blake Barrett, Stephen L. Luther

**Affiliations:** 1 Research & Development Service, James A. Haley Veterans’ Hospital, Tampa, FL USA; 2 Department of Psychology, University of South Florida, Tampa, FL USA; 3 Center of Innovation for Complex Chronic Healthcare (CINCCH), Edward Hines Jr. VA Hospital, Hines, IL USA; 4 VA Center for Clinical Management Research, VA Ann Arbor Healthcare System, Ann Arbor, MI USA; 5 Department of Psychiatry, University of Michigan Medical School, Ann Arbor, MI USA; 6 Department of Environmental Medicine and Public Health, Icahn School of Medicine at Mount Sinai, New York, NY USA; 7 College of Public Health, University of South Florida, Tampa, FL USA; 8 Department of Veterans Affairs VISN 23 Clinical Resource Hub, Minneapolis, MN USA; 9 Mental Health and Behavioral Sciences, James A. Haley Veterans’ Hospital, Tampa, FL USA; 10 Department of Psychiatry and Behavioral Neurosciences, University of South Florida, Tampa, FL USA; 11 Department of Behavioral Sciences and Social Medicine, Florida State University, Tallahassee, FL USA; 12 Mental Health Service, VA Puget Sound Healthcare System at Seattle, Seattle, WA USA

**Keywords:** Depression, Mental health, Treatment initiation, Treatment delay, Classification

## Abstract

**Background:**

Depression is prevalent among Operation Enduring Freedom and Operation Iraqi Freedom (OEF/OIF) Veterans, yet rates of Veteran mental health care utilization remain modest. The current study examined: factors in electronic health records (EHR) associated with lack of treatment initiation and treatment delay; the accuracy of regression and machine learning models to predict initiation of treatment.

**Methods:**

We obtained data from the VA Corporate Data Warehouse (CDW). EHR data were extracted for 127,423 Veterans who deployed to Iraq/Afghanistan after 9/11 with a positive depression screen and a first depression diagnosis between 2001 and 2021. We also obtained 12-month pre-diagnosis and post-diagnosis patient data. Retrospective cohort analysis was employed to test if predictors can reliably differentiate patients who initiated, delayed, or received no mental health treatment associated with their depression diagnosis.

**Results:**

108,457 Veterans with depression, initiated depression-related care (55,492 Veterans delayed treatment beyond one month). Those who were male, without VA disability benefits, with a mild depression diagnosis, and had a history of psychotherapy were less likely to initiate treatment. Among those who initiated care, those with single and mild depression episodes at baseline, with either PTSD or who lacked comorbidities were more likely to delay treatment for depression. A history of mental health treatment, of an anxiety disorder, and a positive depression screen were each related to faster treatment initiation. Classification of patients was modest (ROC AUC = 0.59 95%CI = 0.586–0.602; machine learning F-measure = 0.46).

**Conclusions:**

Having VA disability benefits was the strongest predictor of treatment initiation after a depression diagnosis and a history of mental health treatment was the strongest predictor of delayed initiation of treatment. The complexity of the relationship between VA benefits and history of mental health care with treatment initiation after a depression diagnosis is further discussed. Modest classification accuracy with currently known predictors suggests the need to identify additional predictors of successful depression management.

**Supplementary Information:**

The online version contains supplementary material available at 10.1186/s12913-024-10870-y.

## Background

Depression is highly prevalent worldwide and rates are especially high among Veterans returned from Operation Enduring Freedom and Operation Iraqi Freedom (OEF/OIF), with some reports showing rates as high as nearly 60% [1]. Relative to past cohorts of Veterans, rates of depression in OEF/OIF have also increased over the past decade [2]. To reduce depression-related morbidity and mortality and barriers to care, [3] the Veterans Health Administration (VHA) responded to Veterans’ increasing mental health needs by implementing annual depression screens, embedding psychologists and psychiatrists in primary care to decrease response time, and encouraging same-day referrals for depressed Veterans. Although these changes have increased mental health treatment utilization over time (e.g., from 20% in 2004 to 26% in 2010 utilization of psychotherapy), [4] mental health care utilization for depression among Veterans has remained modest (e.g., 10–26%) [4, 5].

The VA’s substantial efforts to increase access to care underscores a continuing need to address patients’ personal and attitudinal barriers. Specifically, Mojtabai and colleagues [6] highlight the need for a re-evaluation of how we identify patients who do not seek treatment. Negative correlates of receiving mental health treatment over a 12-month period include male gender or being single [7]. Clinical factors have also been implicated in treatment behaviors. For example, Veterans with a history of prior treatment or who have experienced longer, more intense depressive episodes are more likely to seek treatment [8]. 

In this study, we sought to further understand treatment contact correlates in a cohort of OEF/OIF patients with a positive depression screen and a depression diagnosis that received VA services at any time between 2001 and 2021. Our objectives were to: (1) examine factors reliably present in EHR that are associated to lack of treatment initiation and delay in treatment; (2) evaluate the accuracy of proposed models to predict lack of treatment in the future using both regression and machine learning strategies.

## Methods

### Data source and cohort selection

We obtained data from the VA Corporate Data Warehouse (CDW) through the Veterans Affairs Informatics and Computing Infrastructure (VINCI), which contains EHRs of all VHA patients in the United States of America. Inclusion criteria were as follows: Veterans from OEF/OIF cohort between January 2001 – January 2021 (*N* = 1,419,000), with at least one depression diagnosis and one positive depression screen at any time in the study period (*N* = 293,265). Once primary inclusion criteria were applied, patients were excluded for the following reasons: less than 365 days in the VA system before their first depression diagnosis (*n* = 98,984) and if they had received psychotherapy (based on Current Procedural Terminology (CPT) codes) or an antidepressant prescription dispensed the year before their first depression diagnosis (*n* = 51,195) to ascertain that patients were depression treatment free for a full year [9]; fewer than 365 days in the VA system after their first depression treatment or after their last depression diagnosis if no treatment occurred (*n* = 9,561) to allow for at least one full year follow-up period for all patients, and if they had a bipolar, schizophrenia/schizoaffective, or personality disorder diagnosis (*n* = 4,586) or intellectual disabilities or dementia diagnoses (*n* = 1,516) given that all these patients likely went through a different treatment receipt process than the average patient with depression.

### Measures

#### Patient demographics

Extracted CDW data included age, gender, race, Hispanic ethnicity, distance from patient’s residence to the nearest primary, secondary, and tertiary care VA facilities, and rurality or urbanity of patients’ residence. Patient’s VA benefit status was recorded as either being service connected (> 0-100%) or not (0%). Finally, patients’ 12-month healthcare cost incurred by the VA was extracted. All baseline characteristics were anchored on the date of the first depression diagnosis.

*Patient clinical characteristics*: International Classification of Disease, Ninth and Tenth Revision (ICD9/10) codes were extracted to ascertain the presence of at least one depression diagnosis. The first depression diagnosis on file was coded as the index episode marking the baseline of each patient. Episode qualifiers available in the ICD codes were coded into separate variables identifying first (versus recurrent episodes) and mild (versus moderate and severe episodes). ICD codes were also used to extract baseline 12-month comorbidities dating the year prior to the first depression diagnosis: anxiety and adjustment disorders, alcohol and substance disorders, and posttraumatic stress disorder diagnoses.

We employed Nosos scores to risk adjust for clinical co-morbidities [10], computed based on information from the Centers for Medicare and Medicaid Hierarchical Condition Categories (HCC) version-21 using ICD-9 code, age, and gender. The risk scores are then adjusted by incorporating patient pharmacy records and VA-specific factors (e.g., VA priority and costs). The adjusted Nosos score estimates are rescaled to a population mean of one [10]. 

PHQ-9 and PHQ-2 scores obtained during any health care visits at the VA were extracted for the month preceding the first depression diagnosis. Scores were qualified as positive if the PHQ-2 scores were above 2 and the PHQ-9 scores were above 9 [11]. The PHQ-9 is a nine-item instrument that assesses the symptoms of depression corresponding to the Diagnostic and Statistical Manual Version IV (DSM-IV) diagnostic criteria for a major depressive episode and the PHQ-2 is the short version capturing only anhedonia and mood items. Item responses are on a four-point scale (from occurring “not at all” to “nearly every day” over the past two weeks) resulting in a score range from 0 to 27 for the PHQ-9 and 0 to 6 for the PHQ-2.

### Mental health treatment for depression

Treatment *initiation* was measured as at least one completed psychotherapy visit (extracted via CPT codes) associated with a depression diagnosis or a dispensed prescription for any antidepressant within 180 days of a depression diagnosis. This variable was coded as Treatment not initiated = 1 and Treatment initiated = 0.

Treatment *delay* was defined as number of days from date of first depression diagnosis to date of first treatment for depression. Based on VA goals for initiating contact with patients in need of mental healthcare, the continuous variable was transformed into a categorical variable based on the following preset groups coded as: up to 1 week = 6, 1 week − 1 month = 5, 1 month − 3 months = 4, 3 months − 6 months = 3, 6 months − 12 months = 2, and over 1 year = 1.

### Statistical analysis

Descriptive statistics for sociodemographic and clinical characteristics were tabulated for the full cohort of included Veterans. Next, the cohort was randomly split into 70% (*n* = 89,142) testing and 30% (*n* = 38,281) validation participant subsamples to control for spurious findings; effect sizes across variables were less than 0.05 confirming comparability of the randomly created subsamples [12]. 

Univariate and multivariate analyses identified predictors of two outcomes: lack of treatment initiation and treatment initiation delay. Final models were tested using regression adjusting for nested data using Generalized Estimating Equations (GEE) for count, binomial, and multinomial distributions. The best fitting model based on QIC indices was applied to the validation subsample and results are presented in this manuscript. Next, we conducted a series of sensitivity analyses. Specifically, we tested the predictive values of the PHQ-9 score, of a dummy variable identifying those with a positive PHQ-9 score (i.e., PHQ-9 score > 9), reporting any anhedonia, reporting any suicidal ideation. Sensitivity analyses were performed among patients with a PHQ-9 assessment up to a month prior to their depression diagnosis (*n* = 13,610), a timeframe used in other studies to provide a reliable evaluation of the baseline depression severity. All predictors were evaluated based on OR < 0.90 or > 1.1 to avoid multiplicity and OR 95% CI to exclude OR = 1. Given coding, for treatment initiation models ORs < 0.90 reflect odds of initiating treatment and ORs > 1.1 reflect odds of not initiating treatment. Conversely, for treatment delay, ORs < 0.90 reflect odds of longer delay and ORs > 1.1 reflect odds of more timely treatment.

Using the best fitting model, individual Veteran predicted values were saved as the probability of treatment initiation. Based on these probabilities, the area under the receiver operating characteristic (AUC ROC) evaluated the predictive accuracy of the model and determined an optimal cut point to accurately identify patients with depression that would not initiate mental health treatment.

In addition to the GEE regression models, we constructed a machine learning model to perform a multimethod prediction precision evaluation. This study used an updated version of the C4.5 algorithm developed by Quinlan [13] as C5.0. The C5.0 application was compiled from the Global Public License (GPL) C code distributed freely by Quinlan [13]. Specifically, we used decision trees with a 10 × 10 cross validation on the full cohort; we constructed several models with varying values for parameters such as pruning algorithms, minimum number of cases per branch, boosting trials and probabilistic branching. The best model employed pruning based on error rates for each branch, with a minimum of 4 cases for each branch. Boosting and probabilistic branching did not improve the models. We evaluated our model using traditional precision, recall and F measure (see Supplement for additional details).

## Results

### Patient characteristics

See Tables 1 and 2 for the demographic and clinical characteristics of the sample who met inclusion criteria (*N* = 127,423).

**Table 1:** Baseline demographic characteristic at first depression diagnosis ^a^

	Total Sample (*N* = 127,423)	Initiated Depression Treatment (*n* = 108,457)	No Depression Treatment (*n* = 18,966)	Past month PHQ9 (*n* = 13,610)
Age (years)	34.57 (8.88)	34.53 (8.81)	34.84 (9.29)	34.25 (8.80)
Gender (Male)	107,243 (84.2)	90,525 (83.5)	16,718 (88.1)	11,520 (84.6)
Race/Ethnicity				
White non-Hispanic	75,293 (59.1)	63,834 (58.9)	11,459 (60.4)	8045 (59.1)
Black non-Hispanic	25,536 (20.0)	22,059 (20.3)	3477 (18.3)	2821 (20.7)
Hispanic	16,065 (12.6)	13,775 (12.7)	2290 (12.1)	1553 (11.4)
Other	10,529 (8.3)	8789 (8.1)	1740 (9.2)	1191 (8.8)
American Indian/Alaska Native	1483 (1.2)	1279 (1.2)	204 (1.1)	167 (1.2)
Asian	2576 (2.0)	2145 (2.0)	431 (2.3)	279 (2.0)
Native Hawaiian/Other Pacific Islander	1615 (1.3)	1366 (1.3)	249 (1.3)	186 (1.4)
Unknown	8607 (6.8)	7145 (6.6)	1462 (7.7)	977 (7.2)
Marital Status				
Married	61,074 (47.9)	52,143 (48.1)	8931 (47.1)	6470 (47.5)
Divorced/Separated/Widowed	33,598 (26.4)	28,936 (26.7)	4662 (24.6)	3486 (25.6)
Never married	31,733 (24.9)	26,545 (24.5)	5188 (27.4)	3522 (25.9)
Not reported	1018 (0.8)	833 (0.8)	185 (1.0)	132 (1.0)
Driving distance to PC ^b^	15.02 (14.43)	15.03 (14.40)	15.00 (14.56)	15.22 (13.93)
Driving distance to SC ^b^	44.59 (42.26)	44.53 (42.04)	44.88 (43.38)	45.66 (39.72)
Driving distance to TC ^b^	95.01 (135.99)	94.75 (136.48)	96.44 (133.38)	90.14 (113.77)
Rural (Yes)	29,650 (23.3)	25,065 (23.1)	4585 (24.2)	3570 (26.2)
Service connected (Yes)	116,419 (91.4)	100,002 (92.2)	16,417 (86.6)	12,095 (88.9)
Nosos Score (year)	0.57 (0.72)	0.57 (0.74)	0.54 (0.65)	0.50 (0.63)
Health care cost (year)	4756.81 (11322.94)	4820.57 (11675.74)	4434.28 (9329.78)	3815.29 (7480.77)
Total health care visits (year)	34.59 (55.67)	33.39 (53.60)	41.44 (65.84)	25.94 (40.18)

^a^ Reporting mean (SD) for continuous variables and N (%) for categorical variables

^b^ PC = primary care center, SC = secondary care center, TC = tertiary care center

PHQ-9 = Patient Health Questionnaire-9

**Table 2:** Baseline clinical characteristic at first depression diagnosis ^a^

	Total Sample (*N* = 127,423)	Initiated Depression Treatment (*n* = 108,457)	No Depression Treatment (*n* = 18,966)	Past month PHQ9 (*n* = 13,610)
History of MH treatment type b				
None	107,621 (84.5)	92,105 (84.9)	15,516 (81.8)	12,271 (90.2)
Psychotherapy	4172 (3.3)	3308 (3.1)	864 (4.6)	276 (2.0)
Antidepressants	11,113 (8.7)	9367 (8.6)	1746 (9.2)	818 (6.0)
Both	4517 (3.5)	3677 (3.4)	840 (4.4)	245 (1.8)
Comorbid MH disorders				
Adjustment	9217 (7.2)	7993 (7.4)	1224 (6.5)	783 (5.8)
Anxiety	6140 (4.8)	5331 (4.9)	809 (4.3)	515 (3.8)
Alcohol	7037 (5.5)	6105 (5.6)	932 (4.9)	606 (4.5)
Substance	2017 (1.6)	1782 (1.6)	235 (1.2)	152 (1.1)
PTSD	30,165 (23.7)	26,015 (24.0)	4150 (21.9)	2381 (17.5)
No Comorbidity	84,727 (66.5)	71,643 (66.1)	13,084 (69.0)	10,013 (73.6)
Past month depression screen				
Negative PHQ-2 screen	529 (0.4)	469 (0.4)	60 (0.3)	529 (3.9)
Positive PHQ-2 screen	10,915 (8.6)	9192 (8.5)	1723 (9.1)	10,915 (80.2)
PHQ-2 score	4.54 (1.33)	4.57 (1.34)	4.40 (1.26)	4.54 (1.33)
Negative PHQ-9 screen	1757 (1.4)	1438 (1.3)	319 (1.7)	1757 (12.9)
Positive PHQ-9 screen	11,853 (9.3)	10,148 (9.4)	1705 (9.0)	11,853 (87.1)
PHQ-9 score	15.76 (5.3)	15.93 (5.35)	14.73 (5.13)	15.76 (5.33)
Anhedonia item	2.16 (0.92)	2.18 (0.92)	2.09 (0.95)	2.16 (0.92)
Suicide item	0.45 (0.84)	0.47 (0.85)	0.36 (0.78)	0.45 (0.84)
First depression diagnosis				
Mild episode	107,093 (84.0)	90,381 (83.3)	16,712 (88.1)	11,355 (83.4)
Single episode	100,006 (78.5)	84,604 (78.0)	15,402 (81.2)	10,930 (80.3)
Time to depression treatment				
< 1 week	39,798 (31.2)	39,798 (36.7)	-	4522 (33.2)
< 1 month	13,167 (10.3)	13,167 (12.1)	-	1472 (10.8)
< 3 months	18,349 (14.4)	18,349 (16.9)	-	1957 (14.4)
< 6 months	9776 (7.7)	9776 (9.0)	-	1005 (7.4)
< 1 year	6752 (5.3)	6752 (6.2)	-	714 (5.2)
> 1 year	20,615 (16.2)	20,615 (19.0)	-	1916 (14.1)
Never	18,966 (14.9)	-		2024 (14.9)
Depression treatment type initiated				
Psychotherapy	43,386 (34.0)	43,386 (40.0)	-	4882 (35.9)
Antidepressants	60,297 (47.3)	60,297 (55.6)	-	6142 (45.1)
Both	4774 (3.7)	4774 (4.4)	-	562 (4.1)

^a^ Reporting mean (SD) for continuous variables and N (%) for categorical variables

^b^ Psychotherapy or antidepressants received for other disorders than depression

PHQ-9 = Patient Health Questionnaire-9

### Predictors of lack of treatment initiation

Among those patients who met study criteria, 15% (*n* = 18,966) never initiated treatment over the 20-year study period. The odds of not starting treatment increased with lack of service connection, having received psychotherapy in the past, male gender, being never married, and receiving a mild depression diagnosis or other depressive disorder (adjusted ORs > 1.1) (Table 3). Predictors that decreased odds of never starting treatment were having had a past year substance disorder comorbidity (adjusted ORs < 0.9) (Table 3).

**Table 3:** Baseline predictors of lack of treatment initiation for depression among Veterans (validation *n* = 38,281)

	Estimate	Wald Chi-Square	OR ^d^	OR 95%CI
Age (years)	0.003	5.252	1.003	1.000-1.006
Gender (Male)	0.378	86.839	**1.460**	1.348–1.581
Race/Ethnicity				
White non-Hispanic	0	-	1	-
Black non-Hispanic	− 0.086	6.507	0.917	0.859-0.980
Hispanic	− 0.048	1.572	0.953	0.884-1.028
Other ^a^	0.077	3.038	1.080	0.991-1.177
Marital Status				
Married	0	-	1	-
Divorced/Separated/Widowed	− 0.034	1.196	0.967	0.910-1.027
Never married	0.106	11.626	**1.112**	1.046–1.182
Driving distance to SC ^b^	< 0.001	0.985	1.000	1.000-1.001
Rural (Yes)	− 0.048	2.040	0.953	0.892-1.018
Service connected (No)	0.515	208.448	**1.673**	1.560–1.795
NOSOS Score (year)	− 0.048	1.448	0.953	0.881-1.031
Health care cost (year)	< 0.001	0.907	1.000	1.000–1.000
Total health care visits (year)	0.002	91.355	1.002	1.001–1.002
History of MH treatment type ^c^				
None	0	-	1	-
Psychotherapy	0.218	12.856	**1.244**	1.104–1.402
Antidepressants	0.004	0.009	1.004	0.922-1.094
Both	0.003	0.002	1.003	0.876-1.149
Comorbid MH disorders				
Adjustment	− 0.113	2.917	0.893	0.784-1.017
Anxiety	− 0.121	2.991	0.886	0.773-1.016
Alcohol	− 0.043	0.453	0.958	0.845-1.086
Substance	− 0.523	16.850	**0.593**	0.462-0.761
PTSD	− 0.077	1.507	0.926	0.819-1.047
None	− 0.009	0.018	0.991	0.868-1.131
Depression screen				
Negative PHQ-9 screen	0	-	1	-
Positive PHQ-9 screen	− 0.159	2.469	0.853	0.699 − 1.040
PHQ-9 score	− 0.034	17.254	0.967	0.951-0.982
Anhedonia item	0.048	1.071	1.049	0.958-1.148
Suicide item	− 0.033	0.517	0.968	0.884-1.059
First depression diagnosis				
Mild episode	0.269	35.612	**1.309**	1.198–1.430
Single episode	0.064	2.910	1.066	0.990-1.148

^a^ Other = American Indian/Alaska Native, Asian, Native Hawaiian/Other Pacific Islander, Unknown

^b^ SC = secondary care center

^c^ Psychotherapy or antidepressants received for other disorders than depression

^d^ Bold ORs represent significant ORs (< 0.90 or > 1.1 and OR 95%CI ~ = 0)

PHQ-9 = Patient Health Questionnaire-9

### Predictors of delayed treatment

Among those who eventually initiated treatment, 6% (*n* = 6,752) delayed beyond six months and an additional 19% (*n* = 20,615) delayed beyond one year after an initial diagnosis. Clinical predictors such as having a trauma related diagnosis (PTSD, adjustment disorder) or no comorbidities, and a mild or single first depression episode were all significant contributors to delayed treatment initiation. Some factors that contributed to starting treatment earlier rather than later (ORs > 1.1) were in order of importance: having received treatment for depression in the past (psychotherapy and/or antidepressant), not being service connected, or having an anxiety disorder (Table 4). Sensitivity analyses evaluating PHQ9 features as potential predictors, reflected that having a positive depression screen accelerated treatment initiation for depression (OR = 1.294, OR 95%CI = 1.047–1.601).

**Table 4:** Baseline predictors of Veterans delaying* treatment for depression by 1 month to more than 1 year relative to those starting treatment within the first week of a depression diagnosis (validation *n* = 38,281)

	Estimate	Wald-Chi-Square	OR ^d^	OR 95%CI
Age (years)	0.007	34.754	1.007	1.005–1.010
Gender (Male)	0.064	5.369	1.066	1.010–1.125
Race/Ethnicity				
White non-Hispanic	0	-	1	-
Black non-Hispanic	− 0.054	4.114	0.948	0.900-0.998
Hispanic	− 0.012	0.146	0.988	0.931 − 1.050
Other ^a^	− 0.040	1.114	0.961	0.892-1.035
Marital Status				
Married	0	-	1	-
Divorced/Separated/Widowed	0.012	0.230	1.012	0.965-1.061
Never married	− 0.034	1.712	0.966	0.918-1.017
Driving distance to SC ^b^	− 0.001	6.029	0.999	0.999-1.000
Rural (Yes)	− 0.080	8.554	0.923	0.876-0.974
Service connected (No)	0.335	72.651	**1.397**	1.294–1.509
NOSOS Score (year)	− 0.052	3.092	0.949	0.896-1.006
Health care cost (year)	< 0.001	3.403	1.000	1.000–1.000
Total health care visits (year)	< 0.001	1.009	1.000	0.999-1.000
History of MH treatment type ^c^				
None	0	-	1	-
Psychotherapy	0.596	85.265	**1.815**	1.599–2.059
Antidepressants	0.239	40.270	**1.270**	1.180–1.368
Both	0.719	122.326	**2.052**	1.806–2.331
Comorbid disorders				
Adjustment	− 0.165	10.392	**0.848**	0.767-0.937
Anxiety	0.252	21.486	**1.286**	1.156–1.430
Alcohol	0.016	0.096	1.016	0.920-1.122
Substance	− 0.068	0.662	0.934	0.792-1.101
PTSD	− 0.170	12.123	**0.843**	0.766-0.928
None	− 0.184	12.015	**0.832**	0.750-0.923
Depression screen				
Negative PHQ-9 screen	0	-	1	-
Positive PHQ-9 screen	0.258	5.664	**1.294**	1.047–1.601
PHQ-9 score	0.033	15.873	1.033	1.017–1.050
Anhedonia item	− 0.002	0.001	0.998	0.913-1.092
Suicide item	0.009	0.043	1.009	0.930-1.094
First depression diagnosis				
Mild depression episode	− 0.326	101.093	**0.722**	0.677-0.769
Single depression episode	− 0.201	47.528	**0.818**	0.773-0.866

*Treatment delay was coded as 1 = > 1 year, 2 = < 1 year−6 months, 3 = 6−3 months, 4 = 3−1 month, 5 = 1 month−1 week, 6 = < 1 week (reference group). Therefore, ORs < 0.90 reflect odds of higher delay and ORs > 1.1 reflect odds of lower delay

^a^ Other = American Indian/Alaska Native, Asian, Native Hawaiian/Other Pacific Islander, Unknown

^b^ SC = secondary care center

^c^ Psychotherapy or antidepressants received for other disorders than depression

^d^ Bold ORs represent significant ORs (< 0.90 or > 1.1 and OR 95%CI ~ = 0)

PHQ-9 = Patient Health Questionnaire-9

### Identification of patients who never initiated treatment

We tested the accuracy with which we can predict at first diagnosis whether a patient will not start treatment. Our set of demographic and clinical characteristics that a patient presented at their first depression diagnosis led to modest identification of individuals who never initiated depression treatment (AUC = 0.594, 95%CI = 0.586–0.602), given that an AUC of 0.50 is no better than chance.

The resulting machine learning tree was small, with former psychotherapy as the sole predictor. Specifically, patients who received psychotherapy in the past were more likely to never seek treatment again. The overall prediction accuracy of this model was 88.2% on the ten sets of holdout data (Table 5). Despite the high predictive accuracy of this model, its overall prediction of when patients would seek treatment (F-Measure = 0.93) was much better than the prediction of when they do not (F-Measure = 0.46) (Table 5).

**Table 5:** Machine learning prediction precision for treatment initiation

	Initiated Depression Treatment (*n* = 108,457)	No Depression Treatment (*n* = 18,966)
True positive	106,085	6301
False positive	14,712	325
False Negative	325	14,712
Precision	0.87	0.95
Recall	0.99	0.30
F-Measure	0.93	0.46

The precision score was about equal for patients who initiated and did not initiate treatment for depression (0.87 vs. 0.95), indicating that they were effective at distinguishing a true positive from a false positive. With a recall of 0.99, the model was especially effective at identifying patients who eventually seek treatment but very poor at identifying patients who do not (recall = 0.30) (Table 5).

## Discussion

Treatment delays and treatment underutilization for depression remain an all-too-common problem that is associated with increased morbidity and mortality [3]. Our study sought to understand correlates of treatment initiation in a cohort of OEF/OIF Veterans with a depression diagnosis during nearly two decades of VHA services. Our findings provide an initial evaluation of (1) factors associated with absence of treatment for depression or delay in treatment initiation and (2) accuracy of identifying those patients who do not initiate treatment. Our analyses were guided by the larger goal of learning whether routine EHR can be leveraged to develop predictive tools that will identify Veteran treatment choices.

One encouraging finding is that OEF/OIF Veterans with a depression diagnosis initiate mental health care for depression at higher rates than patients in other large health care systems [9]. For example, at three months post initial depression diagnosis, our current cohort showed a nearly 50% higher initiation rate relative to an equally large primary care community cohort (e.g., 35.7%) [9]. This is likely due to the VHA’s efforts to increase same day access for patients with mental health concerns. By a year post initial diagnosis, treatment initiation rate increased by another 50%, so that nearly 70% of our cohort was connecting with mental health care to initiate psychotherapy or antidepressant medication. Our study also found larger initiation rates than other Veteran studies, [4, 5] likely in part due to our evaluation of both antidepressant and psychotherapy initiation, while former studies focused on one treatment type. Still, we found that about one third of patients either took more than a year or never initiated treatment despite receiving a depression diagnosis. This reinforced our effort to understand factors that led to treatment delay, given deleterious effects of deferring treatment for depression [14]. 

### Demographic factors that interfered with receiving treatment or delayed treatment

Our findings aligned with previous work showing that demographic factors play a role in limiting treatment initiation. First, the current study substantiates earlier indications that male gender is often associated with lower odds of receiving mental health treatment [7]. It is possible that acculturation to the military may heighten this possibility. Military training may impact treatment seeking by instilling the value of emotional control while under stress to promote survival and mission completion [15]. These beliefs taken to extreme can promote emotional avoidance and may inadvertently delay treatment seeking [16, 17]. 

Counter to prior findings, [9, 18] our rate of treatment initiation and even delay among Veterans was not related to minority status. This discrepancy may be due to increased access to mental health care among Veterans relative to adults in the general population where access to mental health care is reduced for most minority populations [18]. For example, having insurance/expanded insurance coverage diminished the difference between Latino and non-Latino white populations when evaluating differences in utilization of mental health services [18]. In fact in our study, over 90% of our cohort was service connected for a disability and therefore receiving VA benefits. This factor was the strongest predictor of whether a patient initiated any treatment for depression. While being service connected for a disability was associated with higher rates of treatment initiation, lack of service connection was associated with faster treatment initiation. This may be due to the fact that all Veterans regardless of discharge status which may impact access to VA healthcare, are eligible for one year of mental health services. Therefore, it’s possible that Veterans who may have a more precarious or uncertain access to VA benefits long term, do still take advantage of mental health services that are available and therefore initiate care faster.

### Clinical factors that predict not initiating treatment or delaying treatment

In addition to demographic factors we found that clinical characteristics contributed to lack of treatment or delayed care in three ways.

Firstly, patients were more likely to not receive mental health treatment for depression if their first depressive episode was qualified as mild, unspecified, other, a single episode, or the patient had a past month negative screen, all possibly indicating a recent depression onset. This set of clinical characteristics point to a profile of a less severe, recent diagnosis that may be clinically appropriate for watchful waiting and psychoeducation. Level of illness severity may impact perceived need and thus lead to possible refusal of care; for example, in one study, low perceived need was more often a reason for not seeking treatment among individuals with mild (57.0%) or moderate (39.3%) than severe (25.9%) disorders [6]. 

Secondly, mental health treatment history appeared to differentially impact treatment initiation and treatment delay among those patients who initiated mental health care after a depression diagnosis. Specifically, a history of psychotherapy was both associated with higher odds of never initiating treatment after a depression diagnosis and higher odds of initiating treatment faster if treatment was initiated at all. In the absence of further information about types of treatment and effectiveness, receiving preferred treatment, [19] and therapeutic alliance between provider and patient during treatment [20] in these different groups of patients, it is rather difficult to discern what may have led to this differential impact. Based on prior work there are at least two scenarios for each group. Those patients who never initiated treatment for depression despite a history of psychotherapy, either had a poor experience which led to avoiding psychotherapy despite a new diagnosis or felt confident to reapply strategies learned in the past therapy sessions without initiating a new round of treatment. Patients who reinitiated treatment after a depression diagnosis may have done so quickly because their past experience was successful, or perhaps they were already connected to a provider, [21] or possibly had same day access to services. Further work is needed in this area to more fully understand treatment dynamics over time.

Thirdly, although comorbid substance use disorders were often associated with lack of treatment for depression and PTSD with treatment delay, this may be either because substance use disorders are notoriously associated with low or no treatment initiation [22] or because the VHA has specialty care for both PTSD and substance use disorders readily available, which will likely house most patients with these diagnoses. Although this was not the focus of the current set of analyses, understanding what other mental health services are accessed by patients with depression is an important focus for future work as it has implications for understanding where and whether patients ultimately receive services and support.

### Identification of patients who never started treatment

Finally, the second major goal of this project was to evaluate how predictors of treatment initiation performed in identifying patients who never received treatment. Such work was motivated by our plan to work towards building an automated system that will use EHR data to routinely identify patients at risk of disengaging from care. Our evaluation of the predictive accuracy of our GEE models using AUC ROC and the F-measure for machine learning suggests that using machine learning relative to GEE led to higher accuracy in identifying patients that never started treatment. Specifically, the ratio of true positive to false positive was higher in the machine learning model than the GEE model. Unfortunately, both the GEE prediction precision and the machine learning analysis suggest that the study variables identified and used in our models were insufficient for an accurate decision support tool. Ideally, EHR data may ultimately be used to increase patient engagement with preferred treatments [19]. 

The current study has some limitations. For example, the evaluation of whether treatment is initiated is based on VA services. If patients initiated services outside the VA, these data would not be captured in the current analyses. However, given that over 90% of veterans in this cohort are receiving VA benefits for a disability, they are incentivized to use VA care. Furthermore, the information we used in modeling our outcomes (whether someone defers or delays mental health care) is entirely based on structured data in the CDW. There is clinical data available in clinical notes that may be relevant to the questions raised in this study, but that is more complex to extract. In a recent study we proposed that patients’ affective states may impact how they engage with care and that this information could be extracted from clinical note text reliably [23]. However this line of work is in its infancy and further validation of this data is necessary.

## Conclusions

Future work might consider deriving more targeted variables from EHRs as well as reconsidering key barriers for patients and providers. For example, it is worth investigating how specific debilitating depressive symptoms, like sad mood, [24] influence a Veteran’s decision to seek treatment. Studies have shown that treatment initiation increased with higher depression severity but was only 53% among patients with a PHQ-9 above 9 [9]. Conversely, anhedonia, one of the most prevalent symptoms in depression, [25] was recently related to lower odds of treatment initiation [26]. Finally, providers may also have insights into referral processes that are not evident in structured data from EHRs. This investigation contributes to the field in two ways. First, it reinforces the importance of understanding the barriers and clinical characteristics routinely collected in clinical care, and second, it highlights the need for future work that probes more granularly into clinical characteristics and more specific features that can increase prediction of patients who need support for successful use of VA depression treatment services. Clinically, our work reinforces the importance of providing benefits to Veterans; having adequate health benefits allows patients to engage in care as needed and can have implications for long term depression treatment utilization give heterogeneous trajectories [27]. 

**TABLES**.

### Electronic supplementary material

Below is the link to the electronic supplementary material.

Supplementary Material 1

## Data Availability

The data that support the findings of this study are available from the Veterans Health Administration (VHA) but restrictions apply to the availability of these data, which were used under license for the current study, and so are not publicly available. Data are however available from the authors upon reasonable request and with permission of VHA. Contact Vanessa Panaite, Corresponding Author, with inquiries.

## Abbreviations

AUC ROC: Area Under the Curve Receiver Operating Characteristic
CDW: Corporate Data Warehouse
CI: Confidence Interval
CPT: Current Procedural Terminology
DSM-IV: Diagnostic and Statistical Manual Version IV
EHR: electronic health records
GEE: Generalized Estimating Equations
GPL: Global Public License
HCC: Hierarchical Condition Categories
ICD 9/10: International Classification of Disease, Ninth and Tenth Revision
OEF/OIF: Operation Enduring Freedom and Operation Iraqi Freedom
OR: Odds Ratio
PHQ: Patient Health Questionnaire
PTSD: post traumatic stress disorder
QIC: Quasi Information Criterion
VA: Veterans Administration
VHA: Veterans Health Administration
VINCI: Veterans Affairs Informatics and Computing Infrastructure **Declarations**
