# Supplementary material for: Predictive modeling of initiation and delayed mental health contact for depression
Source: BMC Health Serv Res. 2024 Apr 25;24:529. doi: 10.1186/s12913-024-10870-y (PMC11046938; doi:10.1186/s12913-024-10870-y)
Supplement: Supplementary file 1 — Supplementary Material 1 [file 12913_2024_10870_MOESM1_ESM.docx]

**Supplemental Methods**

**Statistical analysis**

We constructed a machine learning model to perform a multimethod prediction precision evaluation. Since our data consisted of both categorical and continuous variables and a categorical outcome variable, we used decision trees in our analysis. Decision trees were able to generate understandable rules, which can be useful in making inferences about the data. They can perform classification with minimal computation, and they provide a clear indication of which features are most important for prediction.^1^ This study uses an updated version of the C4.5 algorithm developed by Quinlan^2^ as C5.0. The C5.0 application was compiled from the GPL C code distributed freely by Quinlan.^2^

We evaluated our model using traditional precision, recall and F measure. Precision is defined as the fraction of retrieved examples that are relevant and recall is defined as the fraction of relevant examples that are retrieved. Where tp = true positives and fp = false positives, they are calculated by:


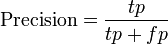

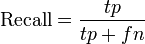


The F-measure (F-Score) combines precision and recall. It is the harmonic mean of precision and recall and is calculated by:


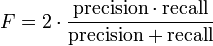


Using 10X10 cross validation on the full cohort, we constructed several models with varying values for parameters such as pruning algorithms, minimum number of cases per branch, boosting trials and probabilistic branching. The best model employed pruning based on error rates for each branch, with a minimum of 4 cases for each branch. Boosting and probabilistic branching did not improve the models.

References:

1. Quinlan JR. Decision trees and decision-making. IEEE Transactions on Systems, Man, and Cybernetics. 1990 Mar;20(2):339-46.
2. Quinlan JR. Rulequest Free Software Downloads. Published online 2013. Accessed August 1, 2013. [ http://www.rulequest.com/download.html]
